# Supplementary material for: Comprehensive evaluation of patterns of hypoglycemia unawareness (HUA) and glycemic variability (GV) in patients with fibrocalculous pancreatic diabetes (FCPD): A cross-sectional study from South India
Source: PLoS One. 2022 Jul 12;17(7):e0270788. doi: 10.1371/journal.pone.0270788 (PMC9275701; doi:10.1371/journal.pone.0270788)
Supplement: S1 Table — (DOCX) [file pone.0270788.s001.docx]

**Supplementary table**

**S1 table: Cardiac autonomic function assessment between groups**

| **Parameters** | **Category** | **Normal: N (%)** | **Abnormal: N (%)** | ***P* value** |
| --- | --- | --- | --- | --- |
| Resting heart rate | HA^*^ (n =12) | 12 (100%) | 0 (0%) | 1.0 |
|  | HU^*^ (n =27) | 23 (85%) | 4 (15%) |  |
| Expiration: Inspiration ratio | HA^*^ (n =12) | 7 (58%) | 5 (42%) | **< 0.01^*^** |
|  | HU^*^ (n =27) | 12 (44%) | 15 (56%) |  |
| Heart Rate on standing | HA^*^ (n =12) | 6 (50%) | 6 (50%) | **< 0.01^*^** |
|  | HU^*^ (n =27) | 6 (26%) | 21 (74%) |  |
| Valsalva manoeuvre | HA^*^ (n =12) | 5 (100%) | 0 (0%) | 1.0 |
|  | HU^*^ (n =27) | 4 (36%) | 7 (64%) |  |
| Blood pressure on standing | HA^*^ (n =12) | 8 (67%) | 4 (33%) | **< 0.01^*^** |
|  | HU^*^ (n =27) | 11 (41%) | 16 (59%) |  |
| Blood pressure on sustained hand grip | HA^*^ (n =12) | 0 (0%) | 12 (100%) | 1.0 |
|  | HU^*^ (n =27) | 0 (0%) | 27 (100%) |  |

Values presented are actual numbers with percentage in parentheses.

p value < 0.05 : Statistically significant ; NS : Statistically significant

HA: Patients with FCPD and hypoglycemia awareness

HU: Patients with FCPD and hypoglycemia unawareness
