# Supplementary material for: Comprehensive evaluation of patterns of hypoglycemia unawareness (HUA) and glycemic variability (GV) in patients with fibrocalculous pancreatic diabetes (FCPD): A cross-sectional study from South India
Source: PLoS One. 2022 Jul 12;17(7):e0270788. doi: 10.1371/journal.pone.0270788 (PMC9275701; doi:10.1371/journal.pone.0270788)
Supplement: S1 File — (DOCX) [file pone.0270788.s002.docx]

**S1 File: Clarke’s questionnaire**:

Survey items used to categorize aware or having reduced awareness of hypoglycemia in subjects

**Check the category that best describes you: ( check one only)**

*a) I always have symptoms when my blood sugar is low (A)*

*b) I sometimes have symptoms when my blood sugar is low (R)*

*c) I no longer have symptoms when my blood sugar is low (*R)

1. **Have you lost some of the symptoms that used to occur when your blood sugar was low?**

*Yes (R) No (A)*

1. **In the past six months how often, have you had moderate hypoglycemia episodes**?

(Episodes where you feel confused, disoriented or lethargic and were unable to treat yourself)

*Never* (A) *Once or twice* (R) *Every other month* (R) *Once a month* (R) *More than once a* *month* (R)

1. **In the past how often you have had severe hypoglycemic episodes** *?*

*(Episodes where you were unconscious or had seizure and needed glucagon and intravenous glucose?)*

Never (A) 1 time (R) 2 times (R) 3 times(R) 4 times(R) 5 times(R) 6 times(R) 7 times(R) 8 times (R) 9 times (R) 10 times (R) 11 times(R) 12 or more times (U)

1. **How often in the last 1 month, you had readings < 70mg/dl with symptoms***?*

1)Never 2)1 to 3 times 3)1 time/week 4) 2 to 3 times/ week 5) 4 to 5 times / week

6) Almost daily

1. **How often you had blood glucose readings < 70mg/dl without symptoms***?*

1)Never 2)1 to 3 times 3)1 time/week 4)2 to 3 times/ week 5) 4 to 5 times / week

6) Almost daily

R= answer to 5 < answer to 6 A= Answer to 6 < answer to 5.

(4 or more R responses = Reduced awareness, 2 or fewer R responses = aware).

1. **How low does your blood sugar needs to go before you feel symptoms?**

60-69mg/dl (A) 50-59mg/dl (A) 40-49mg/dl (R) 40mg/dl (R)

7. **To what extent you can tell by your symptoms that your blood sugar is low?**

Never (R) Rarely (R) Sometimes (R) Often (A) Always (A)

**S2 FILE: Cardiac autonomic function test don**
