# Supplementary material for: Comprehensive evaluation of patterns of hypoglycemia unawareness (HUA) and glycemic variability (GV) in patients with fibrocalculous pancreatic diabetes (FCPD): A cross-sectional study from South India
Source: PLoS One. 2022 Jul 12;17(7):e0270788. doi: 10.1371/journal.pone.0270788 (PMC9275701; doi:10.1371/journal.pone.0270788)
Supplement: S2 File — (DOCX) [file pone.0270788.s003.docx]

**S2 FILE: Cardiac autonomic function test done on CANS 504 ®**

**Parasympathetic function tests :**

1. The heart rate response to deep breathing (E: I ratio) was calculated based on the ratio of the RR interval during deep breathing at six breaths per minute (5 s as inspiration and 5 s as expiration).
2. The heart rate response to standing was calculated between the RR interval at around the 30th beat and the 15th beat from a continuous ECG recording of heart rate for a period 2 min from the supine to the standing position.
3. The heart rate response to Valsalva (Valsalva ratio): the heart rate was recorded at 1 minute before the Valsalva maneouver, during the maneouver and 1 minute following the maneouver. The Valsalva ratio is calculated from the longest RR interval during the phase of relaxation and shortest RR interval during the phase of maneouver.

**Sympathetic function tests:**

1. In the sustained hand grip test, the patient had to squeeze a handgrip dynamometer at a 30% maximum for a period of 5 minutes and an automatic non-invasive blood pressure (NIBP) was measured in the contra-lateral arm. The difference in diastolic blood pressure measured in the contra-lateral arm before the release of contraction and before the beginning of a handgrip procedure was taken as a measure of response.
2. In order to test for a postural drop in blood pressure after standing, the blood pressure was recorded 2 minutes after the patient was supine, and then the patient was made to stand and blood pressure was again checked 2 minutes after the patient was standing. The difference in the systolic blood pressures between supine and standing posture was taken as a measure of response.
