# Supplementary material for: Comprehensive evaluation of patterns of hypoglycemia unawareness (HUA) and glycemic variability (GV) in patients with fibrocalculous pancreatic diabetes (FCPD): A cross-sectional study from South India
Source: PLoS One. 2022 Jul 12;17(7):e0270788. doi: 10.1371/journal.pone.0270788 (PMC9275701; doi:10.1371/journal.pone.0270788)
Supplement: S3 File — (DOCX) [file pone.0270788.s004.docx]

**S3 FILE: Glycemic variability INDICES, ITS expansions and definitions.**

- 1. Standard Deviation (SD): Standard deviation can be calculated as the variability of the glucose levels from the mean glucose value
  2. Continuous Overall Net Glycemic Action (CONGA): It is a continuous glucose monitoring based intraday GV. The Standard deviation (SD) of summated differences between a current observation and observation n hours previously gives the value
  3. Mean Amplitude of Glycemic Excursion (MAGE): It can be calculated from the continuous glucose monitoring – calculated as the difference between the peak and the nadir values of the blood glucose levels.
  4. Average Daily Risk Range (ADRR): It can be calculated as the sum of the daily peak risks for hypoglycemia and hyperglycemia. It is a Composite measure of short-term within-day and between day temporal glucose variability.
  5. Mean of Daily Differences (MODD): It is the 24 hours mean absolute differences between two glucose values measured at the same time point. It can be calculated from the continuous glucose monitoring data.
  6. Time In Range (TIR): It is the percentage of time in which a particular individual’s blood glucose is within the desired target, the target is usually built in the device. TIR for each week can downloaded from the FGMS data and can be compiled together and assessed the mean TIR for each individual.
